# Content Gaps and Informational Quality of TikTok and Bilibili Videos on Pit and Fissure Sealing in China: A Cross-Sectional Study

**DOI:** 10.3290/j.ohpd.c_2759

**Published:** 2026-07-07

**Authors:** Feng Cai, Wending Gao, Linhu Wang

**Affiliations:** a Feng Cai Attending Physician, Department of Stomatology, The Central Hospital of Wuhan, Tongji Medical College, Huazhong University of Science and Technology, Wuhan, China. Conceived the study, screened and assessed videos, analysed the data, and drafted the manuscript.; b Wending Gao Attending Physician, Department of Stomatology, The Central Hospital of Wuhan, Tongji Medical College, Huazhong University of Science and Technology, Wuhan, China. Screened and assessed videos, curated the data, assisted with data analysis, and critically revised the manuscript.; c Linhu Wang Professor, Department of Stomatology, The Central Hospital of Wuhan, Tongji Medical College, Huazhong University of Science and Technology, Wuhan, China. Supervised the study, resolved assessment disagreements, interpreted the data, and critically revised the manuscript.

**Keywords:** Bilibili, oral health communication, pit and fissure sealing, short-video platforms, TikTok

## Abstract

**Purpose:**

To evaluate the disease-specific content coverage, overall quality, reliability, and transparency of Chinese-language short videos on pit and fissure sealing posted on TikTok and Bilibili.

**Methods and Materials:**

In this cross-sectional study, videos were retrieved from TikTok and Bilibili on December 20, 2025, using the Chinese search term ‘窝沟封闭’ (pit and fissure sealing). The first 150 results from each platform were screened, and 204 eligible videos were included. Overall quality, reliability, and transparency were assessed using the Global Quality Score (GQS), modified DISCERN (mDISCERN), and *Journal of the American Medical Association* (JAMA) benchmark criteria, respectively. A predefined disease-specific coding framework was used to evaluate content coverage. Group comparisons, Spearman’s rank correlation, and multivariable ordered logistic regression were performed.

**Results:**

Benefits and indications were the most frequently covered domains, appearing in 87.75% and 78.92% of videos, respectively, whereas contraindications and cost were addressed in only 21.57% and 28.92%. The median GQS, mDISCERN, and JAMA scores were all 3.00. No significant differences in validated assessment scores were observed between TikTok and Bilibili. Videos uploaded by healthcare professionals, especially specialised healthcare professionals, achieved higher scores than those uploaded by individual users. Video duration showed weak positive correlations with validated assessment scores, whereas engagement indicators were not meaningfully associated with informational quality.

**Conclusion:**

Short videos on pit and fissure sealing provide moderately useful information, but content coverage remains incomplete and uneven. Greater participation by healthcare professionals and better access to balanced, trustworthy preventive information are needed on short-video platforms.

Dental caries remains one of the most prevalent chronic diseases in children worldwide and continues to impose a substantial public health burden.^4,18,19,31^ The occlusal surfaces of permanent molars are particularly susceptible to caries because their deep pits and fissures favour plaque retention and are difficult to clean effectively. Pit and fissure sealing is a well-established, minimally invasive preventive intervention that creates a physical barrier over vulnerable tooth surfaces, thereby limiting exposure to cariogenic bacteria and fermentable carbohydrates. It has been shown to reduce occlusal caries and is widely regarded as a cost-effective strategy in public health practice.^2,14,37^ Despite its effectiveness, uptake of pit and fissure sealing remains suboptimal in many settings. Misconceptions among parents, limited awareness of the appropriate timing of treatment, and concerns about material safety may all reduce acceptance of this preventive service.^6,36^ Because parents and caregivers make most decisions regarding children’s preventive dental care, access to accurate and comprehensible information is important for improving oral health literacy, reducing unnecessary concerns, and promoting the use of preventive services, including government-supported programmes where available.^27,35^

With the rapid expansion of digital media, short-video platforms have become increasingly important sources of health information, particularly for parents seeking advice on children’s health issues.^17,38^ In China, TikTok and Bilibili are widely used video-sharing platforms and influential channels for health communication and public health promotion.^7,11^ These platforms are especially relevant in the present context because they are likely to shape how parents and caregivers encounter and interpret information about preventive dental care. For paediatric procedures such as pit and fissure sealing, short videos may communicate indications, procedures, and expected outcomes more intuitively than conventional written materials. However, the open and highly competitive nature of these platforms also raises concerns about the quality of information. Content visibility is often shaped by algorithm-driven content promotion, which may favour novelty, simplicity, or entertainment value over medical completeness and accuracy.^29,32^ As a result, videos may provide incomplete or unbalanced information on clinically important topics, such as indications, contraindications, procedural requirements, follow-up care, material safety, and cost.^29,34^ For a preventive intervention such as pit and fissure sealing, omissions in these areas may influence parental understanding, expectations, and decision-making.

Previous studies evaluating oral health information on social media have reported substantial variation in the quality of content related to orthodontics,^26^ dental implants,^30^ and oral cancer.^3^ However, research on pit and fissure sealing has focused mainly on traditional video platforms such as YouTube,^12^ and evidence from Chinese short-video platforms remains limited. In particular, it remains unclear whether videos on TikTok and Bilibili provide sufficiently comprehensive coverage of clinically important topics, whether their informational quality and reliability are adequate to support informed parental decision-making, and whether these characteristics differ by platform or uploader type. Given the growing role of short-video platforms in health communication in China, addressing these questions is both timely and important.^7,11^ Therefore, the present study evaluated Chinese-language short videos on TikTok and Bilibili about pit and fissure sealing with respect to disease-specific content coverage, overall quality, reliability, and transparency. We hypothesised that these videos would show incomplete coverage in clinically important areas and that videos uploaded by healthcare professionals would achieve higher quality-related scores than those uploaded by individual users.

## METHODS AND MATERIALS

### Data Retrieval and Collection

**Fig 1:**
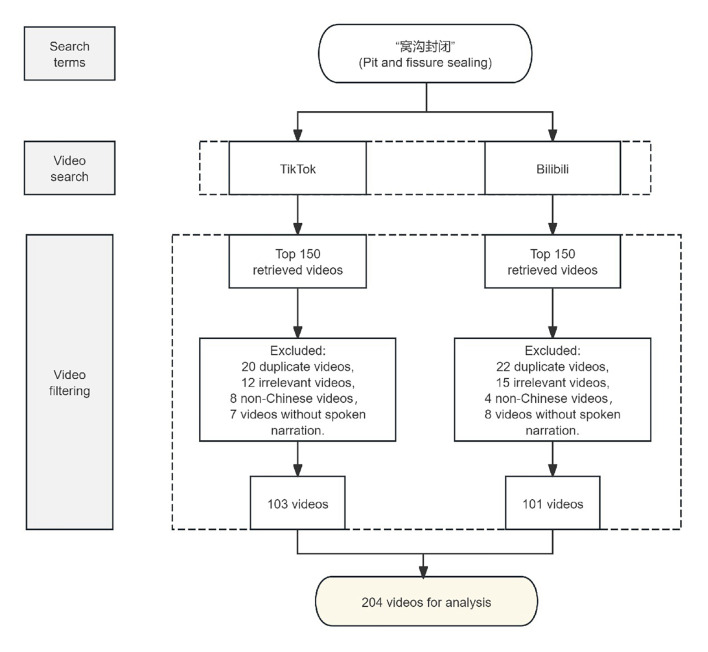
Flow diagram of video identification, screening, and inclusion. Videos on pit and fissure sealing were retrieved from TikTok and Bilibili using the Chinese search term ‘窝沟封闭’ (pit and fissure sealing). The first 150 videos from each platform were screened. Duplicate, irrelevant, non-Chinese, and audio-free videos were excluded. A total of 204 videos were included in the final analysis.

As shown in Figure 1, this cross-sectional study evaluated videos on pit and fissure sealing posted on TikTok and Bilibili. Data were collected on 20 December 2025. On both platforms, the Chinese search term ‘窝沟封闭’ (pit and fissure sealing) was used. For this study, a pit and fissure sealing video was defined as one in which pit and fissure sealing was the primary topic, as determined from the title, audio narration, subtitles, on-screen text, and visual content.

To minimise the influence of personalised recommendation algorithms, all searches were conducted using newly created accounts with no prior browsing history and no previous follows, likes, comments, or other interactions. Browsing history and cache were cleared immediately before data collection. Searches were performed on the desktop web interfaces of TikTok and Bilibili using the default comprehensive ranking mode available at the time of search, defined in this study as the standard algorithm-informed ranking presented to users without the application of additional manual filters. No filters were applied for upload date, popularity, video duration, or uploader type. This approach was intended to approximate the information environment encountered by an ordinary user searching for pit and fissure sealing. Because search outputs on short-video platforms are dynamic and may change rapidly, the retrieved dataset should be interpreted as a time-specific cross-sectional snapshot.

For each platform, the first 150 search results were screened sequentially, starting with the highest-ranked result. This sampling strategy was adopted because videos appearing earlier in search results are more likely to be encountered by users and may therefore exert greater influence on public understanding. In addition, screening the first 150 results from each platform yielded a broad, user-relevant cross-sectional sample while remaining feasible for detailed manual assessment. This approach is broadly consistent with previous cross-sectional studies of health information on TikTok and Bilibili that evaluated top-ranked search results for manual review.^22,28^ No discretionary stopping rules were applied beyond the prespecified sampling frame and eligibility criteria.

Videos were eligible for inclusion if they were presented in Chinese and focused primarily on pit and fissure sealing. Videos were excluded if they were duplicates, unrelated to the study topic, presented in a language other than Chinese, or did not contain spoken audio narration. Videos were considered unrelated when pit and fissure sealing was mentioned only briefly without substantive explanation or when the main content concerned other dental procedures or unrelated oral health topics. Duplicate videos were defined as repeated entries of the same video within the search results of the same platform or substantially identical videos uploaded by the same account on the same platform. In such cases, only one video was retained. Identical or near-identical videos appearing on different platforms were retained because the unit of analysis was platform-specific video exposure. Videos without spoken audio narration were excluded to improve comparability in the assessment of spoken health communication and treatment-related explanation, as several study outcomes required evaluation of explanatory depth, balance, and clinically relevant information commonly conveyed through narration in combination with visual cues. Nevertheless, caption-led or visual-only videos may also convey meaningful health information; this exclusion criterion was therefore recognised as a potential source of selection bias. The detailed identification, screening, and inclusion process is shown in Figure 1.

For each eligible video, the video link, posting date, duration, and engagement indicators were recorded. Engagement indicators included the number of likes, saves, comments, and shares displayed on the platform on the day of data collection. All extracted data were entered into a standardised spreadsheet for subsequent analysis. Save counts were treated as platform-specific indicators of users’ intention to retain or revisit content, although the exact implementation of this function may differ between platforms.

Uploaders were classified into three categories based on platform verification status, account profile information, and other publicly available information: specialised healthcare professionals (SHCPs), non-specialised healthcare professionals (NSHCPs), and individual users (IUs). SHCPs included paediatric dentists, preventive dentistry specialists, and other oral health professionals whose credentials or stated professional roles indicated specialised expertise directly relevant to pit and fissure sealing. NSHCPs included general dentists, nurses, medical institutions, and other healthcare providers without a clearly identifiable speciality in paediatric or preventive oral healthcare. IUs included patients, parents, caregivers, and other laypersons. Uploader classification was performed independently by the two primary assessors, with disagreements resolved by discussion; where necessary, a third senior investigator made the final decision.

### Video Assessment

#### Assessment instruments and content coding

The included videos were evaluated using three established instruments. Overall quality was assessed using the GQS,^5^ reliability using the modified DISCERN (mDISCERN),^9,21^ and transparency using the *Journal of the American Medical Association* (JAMA) benchmark criteria.^33^ These instruments have been widely used in previous studies evaluating online health information.

The GQS is a five-point ordinal scale that evaluates the overall quality, flow, completeness, and usefulness of health information, with scores ranging from 1 (poor) to 5 (excellent). The mDISCERN instrument assesses reliability across five items: clarity and comprehensibility; use of valid sources; balance and lack of bias; provision of additional sources for reference; and discussion of uncertainty. Each item is scored 0 or 1, yielding a total score of 0-5. The JAMA benchmark criteria assess transparency across four domains, namely authorship, attribution, currency, and disclosure. Each domain is scored 0 or 1, yielding a total score of 0–4. For all three instruments, higher scores indicate better quality, reliability, or transparency. Detailed scoring criteria are provided in Supplementary Tables A1 to A3.

In addition to these general assessment tools, disease-specific informational content was evaluated using a coding framework newly developed for this study with reference to relevant professional guidance, including recommendations from the American Dental Association, the American Academy of Pediatric Dentistry,^37^ and the Technical Specifications of the National Children’s Oral Health Comprehensive Intervention Project in China.^25^ The framework was developed to capture clinically important aspects of pit and fissure sealing likely to be addressed in patient- or parent-oriented educational videos. It was refined during pilot calibration before formal coding. It comprised 10 content domains: definition, indications, contraindications, procedures, technique, painlessness, maintenance, safety, benefits, and cost. Operational definitions and coding criteria for each domain are presented in Table 1.

**Table 1:** Disease-specific content coding framework for videos on pit and fissure sealing

Content	Description	Coding criteria (1 = present, 0 = absent)
Definition	Definition of sealant	The video explains what a sealant is (eg, a physical barrier, protective coating, or ‘raincoat’ for the tooth).
Indications	Target population	The video mentions specific indications: deep pits/fissures, first permanent molars (‘six-year molars’), or high caries risk.
Contraindication	Contraindications	The video mentions when NOT to treat: when teeth are not fully erupted (cannot be isolated) or when frank cavitation/decay is present.
Procedures	Workflow overview	The video describes or demonstrates the general steps (cleaning, etching, washing, application, curing).
Technique	Etching and isolation	The video explicitly explains the function of acid etching or emphasises the critical need for moisture control and isolation.
Painlessness	Minimally invasive nature	The video states that the procedure is minimally invasive, painless, and requires no drilling or anaesthesia.
Maintenance	Retention and follow-up	The video mentions the possibility of sealant loss/wear and emphasises the need for periodic recall visits (eg, every 3 or 6 months).
Safety	Material safety	The video addresses the safety of the materials used (eg, non-toxic, resin-based, safe for children).
Benefits	Efficacy	The video explicitly states that sealants are effective in preventing dental caries (cavities).
Cost	Cost or policy	The video discusses the cost of treatment or mentions government-sponsored free sealant programmes.
Presence of each disease-specific content item was coded as 1, and absence was coded as 0.		

Each domain was coded as present or absent. A domain was considered present when the video explicitly mentioned, explained, or clearly demonstrated the corresponding topic, as defined by the predefined criteria. Because a single video could address multiple domains, all domains were coded independently and were not mutually exclusive. The coding framework was used primarily to describe the distribution and coverage of disease-specific information. For sensitivity analysis, an unweighted content completeness score was also calculated as the sum of the 10 domains, ranging from 0 to 10.

#### Assessment procedure

Before formal assessment, the two primary assessors (FC and WG) independently reviewed the scoring criteria for GQS, mDISCERN, and the JAMA benchmark criteria, as well as the disease-specific coding framework. A training session was then conducted to promote consistent interpretation of each item in the context of Chinese-language short videos. For videos containing personal narratives, entertainment elements, or informal expressions, assessors were instructed to focus on extractable health-related information relevant to pit and fissure sealing.

A pilot assessment was conducted on a random sample of 20 videos to calibrate coding and scoring procedures. Interrater agreement during the pilot phase was assessed using Cohen’s kappa and showed good agreement (κ = 0.84). After calibration, all included videos were independently assessed by the two primary assessors. Disagreements in uploader classification, content coding, or instrument scoring were resolved through discussion; if consensus could not be reached, a third senior investigator (LW) made the final decision.

To reduce assessment bias, engagement metrics were not used during scoring. Assessors evaluated each video based on the information presented through narration, subtitles, on-screen text, and visual demonstrations. All ratings and coding decisions were recorded in a standardised spreadsheet.

### Ethics Statement

Because this study analysed publicly accessible online videos and did not involve interaction with human participants or the collection of identifiable private information, formal ethics committee approval and informed consent were not required under the policy of The Central Hospital of Wuhan, China.

### Statistical Analysis

Continuous variables were assessed for normality using the Shapiro-Wilk test. As these variables were not normally distributed, they are presented as medians with interquartile ranges. Categorical variables are presented as frequencies and percentages. Video characteristics, engagement indicators, assessment scores, and disease-specific content coverage were summarised for the overall sample and stratified by platform and uploader category.

Comparisons between TikTok and Bilibili were performed using the Mann–Whitney U test for continuous variables and the Pearson chi-square test or Fisher’s exact test for categorical variables, as appropriate. Comparisons among uploader categories were performed using the Kruskal–Wallis test for continuous variables and the Pearson chi-square test or Fisher’s exact test for categorical variables, as appropriate. When the Kruskal–Wallis test indicated a significant overall difference, post hoc pairwise comparisons were conducted using Dunn’s test with Bonferroni-adjusted *P* values.

Spearman’s rank correlation analysis was used to examine associations among video duration, engagement indicators, and assessment scores, including duration, likes, saves, comments, shares, GQS, mDISCERN, and JAMA scores.

To identify factors associated with higher video quality, multivariable ordered logistic regression was performed with GQS category as the ordinal dependent variable. GQS was analysed as an ordinal outcome because it represents ordered categories of overall quality. The primary model included platform, uploader category, natural log-transformed video duration, and natural log-transformed total engagement plus 1 as independent variables. Total engagement was defined as the sum of likes, saves, comments, and shares to reflect overall user interaction, although these indicators may capture different forms of engagement. Engagement and duration variables were log-transformed because their distributions were markedly right-skewed, thereby reducing skewness and limiting the influence of extreme values. Bilibili and IUs were used as the reference categories for the platform and uploader categories, respectively. A sensitivity analysis was additionally performed by including the unweighted content completeness score.

Before model fitting, multicollinearity among independent variables was assessed using variance inflation factors, and the proportional odds assumption was evaluated using the Brant test. Adjusted odds ratios with 95% confidence intervals were reported.

All statistical analyses were performed using R software, version 4.3.3. All tests were two-sided, and P values below 0.05 were considered statistically significant. Given the exploratory nature of the study, findings from multiple comparisons were interpreted cautiously.

## RESULTS

### Search Results and Video Characteristics

Using the predefined search strategy, the first 150 results from each platform were screened, yielding a total of 300 videos. After applying the inclusion and exclusion criteria, 204 videos were included in the final analysis, comprising 103 TikTok videos (50.49%) and 101 Bilibili videos (49.51%) (Fig 1).

As shown in Table 2, most videos were uploaded by NSHCPs, accounting for 107 of 204 (52.45%), followed by IUs with 76 (37.25%) and SHCPs with 21 (10.29%). The median video duration was 73.50 s (Q1, Q3: 54.00, 131.00). Median engagement metrics were 21.00 likes (Q1, Q3: 5.75, 100.00), 55.00 saves (Q1, Q3: 8.00, 158.00), 2.50 comments (Q1, Q3: 0.00, 16.25), and 7.50 shares (Q1, Q3: 2.00, 47.75). The median GQS, mDISCERN, and JAMA scores were all 3.00, indicating a moderate overall level of quality, reliability, and transparency.

**Table 2:** Overall video characteristics

Characteristics	Total (n = 204)
**Short-video platforms n (%)**	
TikTok	103 (50.49%)
Bilibili	101 (49.51%)
**Video source n (%)**	
SHCPs	21 (10.29%)
NSHCPs	107 (52.45%)
IUs	76 (37.25%)
**General information**	
Duration (s), Median (Q1, Q3)	73.50 (54.00, 131.00)
Likes, Median (Q1, Q3)	21.00 (5.75, 100.00)
Saves, Median (Q1, Q3)	55.00 (8.00, 158.00)
Comments, Median (Q1, Q3)	2.50 (0.00, 16.25)
Shares, Median (Q1, Q3)	7.50 (2.00, 47.75)
**Video quality**	
GQS, Median (Q1, Q3)	3.00 (3.00, 4.00)
mDISCERN, Median (Q1, Q3)	3.00 (3.00, 4.00)
JAMA, Median (Q1, Q3)	3.00 (3.00, 4.00)
**Video content n (%)**	
Definition	156 (76.47%)
Indications	161 (78.92%)
Contraindications	44 (21.57%)
Procedures	91 (44.61%)
Technique	78 (38.24%)
Painlessness	70 (34.31%)
Maintenance	83 (40.69%)
Safety	119 (58.33%)
Benefits	179 (87.75%)
Cost	59 (28.92%)
Data are presented as n (%) or median (Q1, Q3), as appropriate. Percentages for specific content coverage were calculated based on the total sample (n = 204). SHCPs, specialised healthcare professionals; NSHCPs, non-specialised healthcare professionals; IUs, individual users; GQS, Global Quality Score; mDISCERN, modified DISCERN; JAMA, *Journal of the American Medical Association* benchmark criteria.	

**Fig 2:**
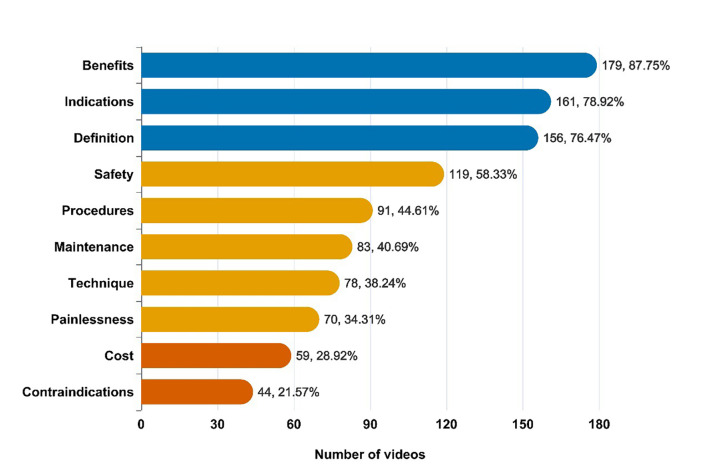
Coverage of disease-specific content domains in videos on pit and fissure sealing. The bar chart shows the number and percentage of videos covering each predefined content domain, including benefits, indications, definition, safety, procedures, maintenance, technique, painlessness, cost, and contraindications. Percentages were calculated based on the total sample of 204 videos. Bars were colour-coded according to coverage level: high coverage (≥ 60%; blue), moderate coverage (30–59%; orange), and low coverage (< 30%; vermilion).

The distribution of disease-specific content is shown in Figure 2 and Table 2. Benefits were the most frequently covered domain, appearing in 179 of 204 videos (87.75%), followed by indications in 161 videos (78.92%) and definition in 156 videos (76.47%). Safety was mentioned in 119 videos (58.33%). By contrast, procedures, maintenance, technique, painlessness, and cost were addressed less frequently, with coverage ranging from 28.92% to 44.61%. Contraindications were the least frequently covered domain, appearing in only 44 videos (21.57%). Overall, the videos tended to emphasise the benefits, indications, and basic concept of pit and fissure sealing. In contrast, clinically relevant topics such as contraindications, technical details, follow-up maintenance, and cost were discussed far less often.

### Comparison of Video Characteristics between Platforms

Detailed comparisons between TikTok and Bilibili are presented in Table 3. Bilibili videos were significantly longer than TikTok videos, with a median duration of 109.00 s (Q1, Q3: 66.00, 156.00) compared with 63.00 s (Q1, Q3: 52.50, 93.00) (*P* < 0.001). In contrast, TikTok videos showed higher levels of observable user engagement. Median numbers of likes, comments, and shares were 58.00, 8.00, and 13.00, respectively, on TikTok, compared with 7.00, 1.00, and 4.00 on Bilibili (all *P* < 0.001). However, Bilibili videos received significantly more saves than TikTok videos, with median values of 78.00 and 12.00, respectively (*P* < 0.001).

**Table 3:** Comparison of video characteristics, assessment scores, and specific content coverage by platform

Variables	TikTok (n = 103)	Bilibili (n = 101)	*P* value
**General information and engagement**			
Duration, Median (Q1, Q3)	63.00 (52.50, 93.00)	109.00 (66.00, 156.00)	< 0.001
Likes, Median (Q1, Q3)	58.00 (16.00, 248.50)	7.00 (3.00, 30.00)	< 0.001
Saves, Median (Q1, Q3)	12.00 (4.00, 90.50)s	78.00 (31.00, 456.00)	< 0.001
Comments, Median (Q1, Q3)	8.00 (2.00, 30.50)	1.00 (0.00, 4.00)	< 0.001
Shares, Median (Q1, Q3)	13.00 (3.00, 174.50)	4.00 (1.00, 23.00)	< 0.001
**Quality and reliability scores**			
GQS, Median (Q1, Q3)	3.00 (3.00, 4.00)	3.00 (2.00, 4.00)	0.266
mDISCERN, Median (Q1, Q3)	3.00 (3.00, 4.00)	3.00 (2.00, 4.00)	0.434
JAMA, Median (Q1, Q3)	3.00 (3.00, 4.00)	3.00 (2.00, 3.00)	0.216
**Specific content coverage, n (%)**			
Definition	77 (74.76%)	79 (78.22%)	0.676
Indications	85 (82.52%)	76 (75.25%)	0.270
Contraindications	19 (18.45%)	25 (24.75%)	0.355
Procedures	43 (41.75%)	48 (47.52%)	0.491
Technique	33 (32.04%)	45 (44.55%)	0.090
Painlessness	36 (34.95%)	34 (33.66%)	0.963
Maintenance	42 (40.78%)	41 (40.59%)	1.000
Safety	57 (55.34%)	62 (61.39%)	0.463
Benefits	89 (86.41%)	90 (89.11%)	0.708
Cost	42 (40.78%)	17 (16.83%)	< 0.001
Data are presented as n (%) or median (Q1, Q3), as appropriate. Continuous variables were compared using the Mann–Whitney U test, and categorical variables were compared using the Pearson chi-square test or Fisher’s exact test, as appropriate. GQS, Global Quality Score; mDISCERN, modified DISCERN; JAMA, *Journal of the American Medical Association* benchmark criteria.			

No significant differences in validated assessment scores were observed between platforms. The distributions of GQS, mDISCERN, and JAMA scores were comparable for TikTok and Bilibili (GQS: *P* = 0.266; mDISCERN: *P* = 0.434; JAMA: *P* = 0.216). Disease-specific content coverage was also broadly similar across platforms. Among the 10 content domains, only cost differed significantly, being discussed more frequently in TikTok videos than in Bilibili videos (40.78% vs 16.83%, *P* < 0.001). Although the technique was mentioned more often in Bilibili videos than in TikTok videos (44.55% vs 32.04%), this difference did not reach statistical significance (*P* = 0.090). No significant between-platform differences were found for the remaining content domains.

### Comparison of Video Characteristics by Uploader Category

Comparisons across uploader categories are summarised in Table 4. Video duration differed significantly among SHCPs, NSHCPs, and IUs (*P* = 0.002). Videos uploaded by SHCPs were the longest, with a median duration of 135.00 s (Q1, Q3: 82.00, 224.00), followed by NSHCP-uploaded videos at 76.00 s (Q1, Q3: 57.00, 124.00) and IU-uploaded videos at 66.00 s (Q1, Q3: 47.00, 109.00). By contrast, engagement indicators, including likes, saves, comments, and shares, did not differ significantly across uploader categories (all *P* > 0.05).

**Table 4:** Comparison of video characteristics, assessment scores, and specific content coverage by uploader category

Variables	SHCPs (n = 21)	NSHCPs (n = 107)	IUs (n = 76)	*P* value
**General information and engagement**				
Duration, Median (Q1, Q3)	135.00 (82.00, 224.00)	76.00 (57.00, 124.00)	66.00 (47.00, 109.00)	0.002
Likes, Median (Q1, Q3)	30.00 (8.00, 97.00)	27.00 (5.50, 115.00)	14.00 (4.75, 76.25)	0.557
Saves, Median (Q1, Q3)	90.00 (11.00, 482.00)	44.00 (8.00, 97.00)	56.00 (7.75, 456.00)	0.588
Comments, Median (Q1, Q3)	3.00 (1.00, 16.00)	3.00 (1.00, 16.00)	1.00 (0.00, 16.25)	0.446
Shares, Median (Q1, Q3)	6.00 (3.00, 51.00)	7.00 (2.00, 39.00)	11.50 (2.00, 57.00)	0.646
**Quality and reliability scores**				
GQS, Median (Q1, Q3)	4.00 (3.00, 5.00)	3.00 (3.00, 4.00)	2.00 (2.00, 3.00)	< 0.001
mDISCERN, Median (Q1, Q3)	4.00 (4.00, 5.00)	4.00 (3.00, 4.00)	2.00 (2.00, 3.00)	< 0.001
JAMA, Median (Q1, Q3)	4.00 (3.00, 4.00)	3.00 (3.00, 4.00)	2.00 (2.00, 3.00)	< 0.001
**Specific content coverage, n (%)**				
Definition	16 (76.19%)	86 (80.37%)	54 (71.05%)	0.342
Indications	18 (85.71%)	89 (83.18%)	54 (71.05%)	0.101
Contraindications	8 (38.10%)	26 (24.30%)	10 (13.16%)	0.030
Procedures	14 (66.67%)	52 (48.60%)	25 (32.89%)	0.011
Technique	10 (47.62%)	43 (40.19%)	25 (32.89%)	0.392
Painlessness	13 (61.90%)	40 (37.38%)	17 (22.37%)	0.002
Maintenance	16 (76.19%)	49 (45.79%)	18 (23.68%)	< 0.001
Safety	18 (85.71%)	73 (68.22%)	28 (36.84%)	< 0.001
Benefits	18 (85.71%)	95 (88.79%)	66 (86.84%)	0.884
Cost	5 (23.81%)	32 (29.91%)	22 (28.95%)	0.853
Data are presented as n (%) or median (Q1, Q3), as appropriate. Continuous variables were compared using the Kruskal–Wallis test, and categorical variables were compared using the Pearson chi-square test or Fisher’s exact test, as appropriate. Post hoc pairwise comparisons for GQS, mDISCERN, and JAMA were performed using Dunn’s multiple-comparisons test with adjusted *P* values. SHCPs, specialised healthcare professionals; NSHCPs, non-specialised healthcare professionals; IUs, individual users; GQS, Global Quality Score; mDISCERN, modified DISCERN; JAMA, *Journal of the American Medical Association* benchmark criteria.				

**Fig 3a to c:**
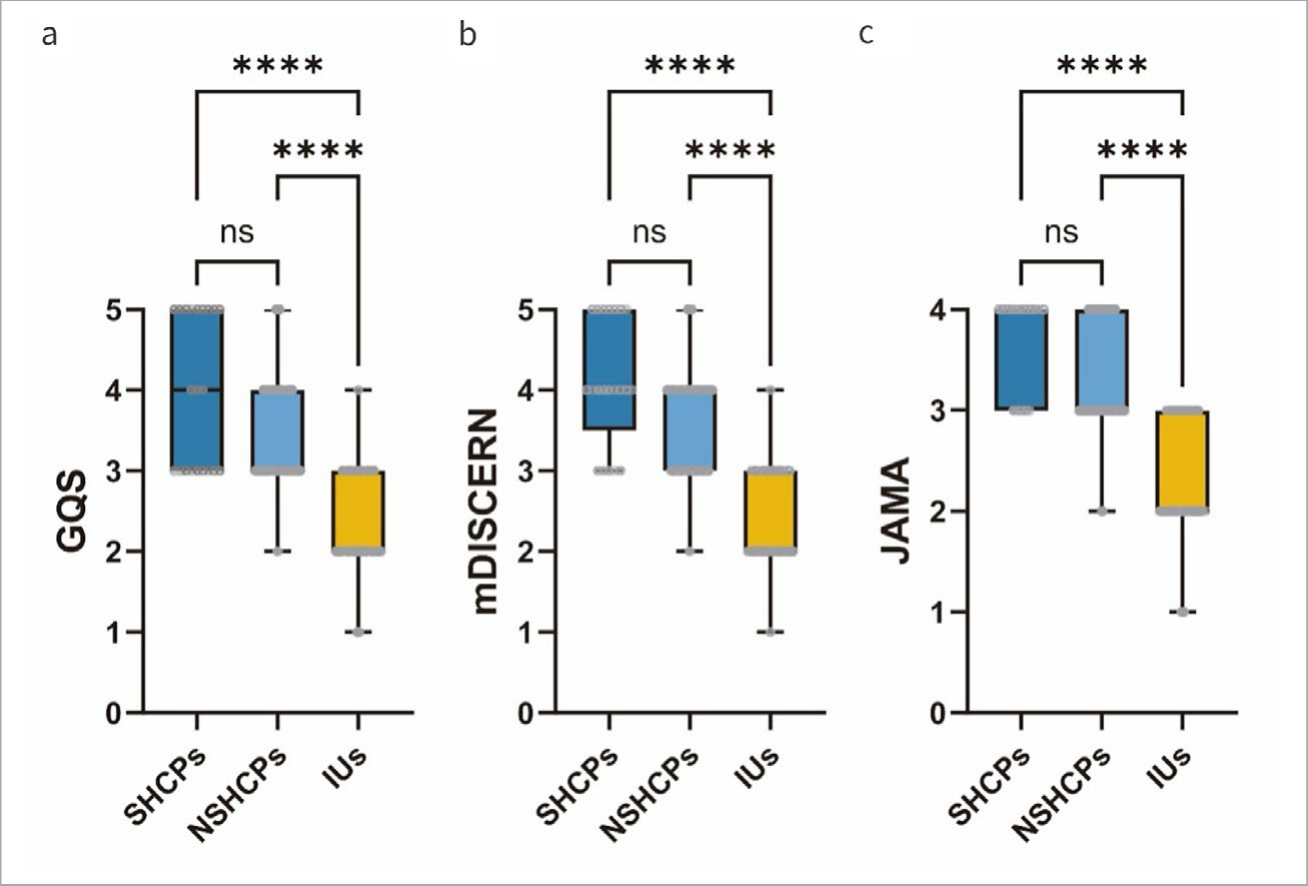
Comparison of video assessment scores by uploader category. Box-and-whisker plots show the distributions of (a) Global Quality Score (GQS), (b) modified DISCERN (mDISCERN), and (c) JAMA benchmark scores across videos uploaded by specialised healthcare professionals (SHCPs), non-specialised healthcare professionals (NSHCPs), and individual users (IUs). Boxes represent the interquartile range, centre lines indicate the median, whiskers indicate the minimum and maximum values, and points represent individual videos. Overall group differences were assessed using the Kruskal–Wallis test, followed by Dunn’s multiple comparisons test with adjusted *P* values for pairwise comparisons. ns, not significant; **** *P* < 0.0001.

Marked differences were observed in validated assessment scores. Median GQS, mDISCERN, and JAMA scores all differed significantly among uploader categories (all *P *< 0.001). SHCP-uploaded videos had the highest median scores for GQS [4.00 (3.00, 5.00)], mDISCERN [4.00 (4.00, 5.00)], and JAMA [4.00 (3.00, 4.00)], whereas IU-uploaded videos had the lowest corresponding scores [2.00 (2.00, 3.00), 2.00 (2.00, 3.00), and 2.00 (2.00, 3.00), respectively]. NSHCP-uploaded videos showed intermediate performance. Post hoc pairwise comparisons indicated no significant differences between SHCPs and NSHCPs, whereas both groups scored significantly higher than IUs across all three assessment tools (Fig 3).

Differences in disease-specific content coverage were also observed across uploader categories. Significant between-group differences were found for contraindications (*P* = 0.030), procedures (*P* = 0.011), painlessness (*P* = 0.002), maintenance (*P* < 0.001), and safety (*P* < 0.001). Overall, videos uploaded by SHCPs were more likely to address these clinically relevant domains than those uploaded by NSHCPs and IUs. For example, maintenance was covered in 76.19% of SHCP videos, compared with 45.79% of NSHCP videos and 23.68% of IU videos. Safety was discussed in 85.71% of SHCP videos, 68.22% of NSHCP videos, and 36.84% of IU videos. Likewise, painlessness was mentioned in 61.90% of SHCP videos, compared with 37.38% of NSHCP videos and 22.37% of IU videos.

No significant differences across uploader categories were found for definition, indications, technique, benefits, or cost (all *P* > 0.05). Taken together, videos uploaded by healthcare professionals, particularly SHCPs, tended to be longer, achieved higher validated assessment scores, and more often covered clinically relevant aspects of pit and fissure sealing than those uploaded by IUs.

### Correlation Analysis of Video Characteristics, Engagement Metrics, and Assessment Scores

**Fig 4:**
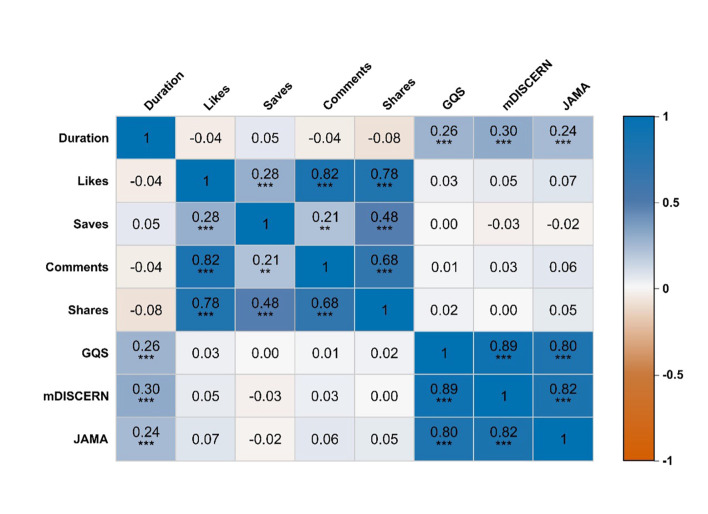
Spearman correlation matrix of video characteristics, engagement metrics, and assessment scores. The heatmap shows the pairwise Spearman rank correlation coefficients among video duration, engagement metrics (likes, saves, comments, and shares), and assessment scores (GQS, mDISCERN, and JAMA) across the 204 included videos. Blue indicates positive correlations and orange indicates negative correlations, with darker shades representing stronger associations. Asterisks indicate statistical significance (*P < 0.05; ** *P* < 0.01; *** *P* < 0.001). GQS, Global Quality Score; mDISCERN, modified DISCERN; JAMA, *Journal of the American Medical Association* benchmark criteria.

The correlations among video duration, engagement metrics, and assessment scores are shown in Figure 4. Video duration was weakly but significantly positively correlated with GQS (Spearman ρ = 0.26, *P* < 0.001), mDISCERN (Spearman ρ = 0.30, *P* < 0.001), and JAMA (Spearman ρ = 0.24, *P* < 0.001), indicating that longer videos tended to receive higher quality, reliability, and transparency scores.

By contrast, user engagement indicators were not significantly associated with the validated assessment scores. Likes, saves, comments, and shares all showed negligible correlations with GQS, mDISCERN, and JAMA, suggesting that more popular or more interactive videos were not necessarily of higher informational quality.

The three assessment instruments were strongly positively correlated with one another. GQS was strongly correlated with mDISCERN (Spearman ρ = 0.89, *P* < 0.001) and JAMA (Spearman ρ = 0.80, *P* < 0.001), and mDISCERN was also strongly correlated with JAMA (Spearman ρ = 0.82, *P* < 0.001). These findings indicate substantial concordance among the three instruments in identifying videos with higher informational value.

Engagement metrics were positively correlated with one another. In particular, likes were strongly correlated with comments (Spearman’s ρ = 0.82, *P* < 0.001) and shares (Spearman’s ρ = 0.78, *P* < 0.001), and comments were also positively correlated with shares (Spearman’s ρ = 0.68, *P* < 0.001). Saves showed moderate positive correlations with shares (Spearman’s ρ = 0.48, *P* < 0.001) and likes (Spearman’s ρ = 0.28, *P* < 0.001), and a weaker but still significant correlation with comments (Spearman’s ρ = 0.21, *P* = 0.003). Duration was not meaningfully correlated with the engagement indicators. Overall, these findings suggest that viewer interaction primarily reflects platform engagement behaviour rather than informational quality or reliability.

### Factors Associated with Higher GQS Categories

The multivariable ordered logistic regression analysis is presented in Table 5. After adjustment for platform, uploader category, video duration, and total engagement, uploader category remained strongly associated with higher GQS categories. Compared with videos uploaded by IUs, those uploaded by NSHCPs had substantially higher odds of being classified in a higher GQS category (adjusted OR = 67.97, 95% CI: 19.82 to 233.06, *P* < 0.001), and the association was even stronger for SHCP-uploaded videos (adjusted OR = 224.09, 95% CI: 49.21 to 1020.43, *P* < 0.001).

**Table 5:** Multivariable ordered logistic regression analysis of factors associated with higher GQS categories

Variables	Adjusted OR (95% CI)	*P* value	Sensitivity analysis, OR (95% CI)	*P* value
**Platform**				
Bilibili	Ref		Ref	
TikTok	1.28 (0.72, 2.30)	0.404	1.37 (0.73, 2.58)	0.332
**Uploader category**				
IUs	Ref		Ref	
NSHCPs	67.97 (19.82, 233.06)	< 0.001	51.95 (14.49, 186.23)	< 0.001
SHCPs	224.09 (49.21, 1020.43)	< 0.001	112.79 (22.77, 558.61)	< 0.001
In (Duration)	1.71 (1.10, 2.67)	0.017	1.00 (0.61, 1.63)	0.999
In (Total engagement + 1)	1.04 (0.92, 1.18)	0.496	1.11 (0.97, 1.27)	0.138
Content completeness score (per 1-point increase)	–	–	2.91 (2.20, 3.85)	< 0.001
Higher GQS categories were modelled as the ordered outcome. ORs > 1 indicate higher odds of being in a higher GQS category. In (Duration) and In (Total engagement + 1) denote natural log-transformed variables. Total engagement was calculated as the sum of likes, saves, comments, and shares. The reference categories were Bilibili for the platform and IUs for the uploader category. SHCPs, specialised healthcare professionals; NSHCPs, non-specialised healthcare professionals; IUs, individual users; OR, odds ratio; CI, confidence interval. A sensitivity analysis additionally included the unweighted content completeness score, calculated as the sum of 10 predefined content domains (range 0–10).				

Longer video duration was also independently associated with higher GQS categories in the primary model (adjusted OR = 1.71, 95% CI: 1.10 to 2.67, *P* = 0.017). By contrast, platform was not significantly associated with GQS category (TikTok vs Bilibili: adjusted OR = 1.28, 95% CI: 0.72 to 2.30, *P* = 0.404), nor was total engagement (adjusted OR = 1.04, 95% CI: 0.92 to 1.18, *P* = 0.496).

In the sensitivity analysis, which additionally included the unweighted content completeness score, content completeness was strongly associated with higher GQS categories (adjusted OR = 2.91, 95% CI: 2.20 to 3.85, *P* < 0.001). The associations for the uploader category remained robust, whereas the association between video duration and the GQS category was attenuated and no longer statistically significant (adjusted OR = 1.00, 95% CI: 0.61 to 1.63, *P* = 0.999). These findings suggest that the uploader’s background and the completeness of disease-specific information were the main factors associated with higher overall video quality.

## DISCUSSION

### Principal Findings

This study evaluated TikTok and Bilibili videos on pit and fissure sealing for disease-specific content coverage, overall quality, reliability, and transparency. Several findings merit emphasis. First, the video information profiles were incomplete. Most videos focused on the benefits, indications, and basic concept of pit and fissure sealing, whereas contraindications, technical considerations, follow-up maintenance, and cost were addressed much less frequently. Second, the uploader category was strongly associated with video quality.

Videos uploaded by healthcare professionals, particularly SHCP, consistently achieved higher GQS, mDISCERN, and JAMA scores than those uploaded by IUs. Third, engagement metrics were not associated with validated quality measures, indicating that popularity did not reflect informational value. Finally, although longer videos tended to have higher assessment scores in univariable analyses, this association attenuated after adjustment for content completeness, suggesting that informational breadth may be more important than duration alone.

Taken together, these findings suggest that short-video platforms may increase public exposure to preventive oral health topics, but they do not necessarily provide balanced or decision-relevant information for parents and caregivers. This issue is particularly important in paediatric oral health, where caregiver knowledge and expectations may influence the uptake of preventive services.

### Video Content, Uploader Source, and Informational Quality

The content pattern observed in this study warrants attention. Most videos explained what pit and fissure sealing is and emphasised its preventive benefits, but far fewer addressed contraindications, follow-up maintenance, technical requirements, or cost. This selective presentation is broadly consistent with the communication style of short-form video platforms, where concise and affirmative messages are more likely to attract attention. However, decisions about pit and fissure sealing require more than general awareness. Parents and caregivers also need to understand when sealing may not be appropriate, why isolation and technique matter, whether retention requires periodic review, and how services can be accessed in practice.

The low frequency of maintenance-related information is clinically relevant. Pit and fissure sealing should not be viewed as a one-time procedure that ends at placement. Its preventive effect depends on retention, periodic review, and timely repair or replacement when necessary. Likewise, the limited discussion of contraindications may leave viewers with an overly simplified impression that sealing is universally appropriate. In preventive care, incomplete information may influence decision-making even when the core message is not factually incorrect.

The limited mention of cost is also notable in the Chinese context. Public oral health programmes and population-based studies in China have shown that pit and fissure sealing is an established component of childhood caries prevention and can achieve measurable anticaries benefits in school-aged children.^23,24^ Against this background, the relatively infrequent mention of school-based, public, or subsidised sealant services may weaken the link between online health education and actual service use.

Uploader source was another important determinant of information quality. In the present study, videos uploaded by SHCP achieved the highest median scores, and both specialised and NSHCP outperformed IUs on validated quality measures. Healthcare professional uploaders were also more likely to address clinically relevant topics such as contraindications, procedures, painlessness, maintenance, and safety. These descriptive findings were reinforced by the multivariable analysis, in which uploader category remained strongly associated with higher GQS categories after adjustment for platform, duration, and engagement. This pattern is consistent with previous studies showing that professionally produced online health information tends to perform better on validated quality assessments than user-generated content.^10,22^ Research on oral health videos has similarly reported uneven quality and a mismatch between popularity and reliability across video-sharing platforms.^12,15,20^

Platform-related differences should be interpreted cautiously. Bilibili videos were significantly longer and received more saves, whereas TikTok videos generated more likes, comments, and shares. However, no significant between-platform differences were observed in GQS, mDISCERN, or JAMA scores, and platform was not independently associated with higher GQS categories in the multivariable model. These findings suggest that platform format may shape patterns of user interaction, but not necessarily the underlying informational quality. Longer duration may allow more room for explanation, yet duration alone does not guarantee completeness or reliability. Similarly, higher visible engagement does not imply greater educational value.

### Comparison with Previous Research and Interpretive Implications

GQS, mDISCERN, and JAMA are widely used instruments for evaluating online health information and provide complementary perspectives on usefulness, reliability, and transparency.^13,29^ Their combined use in the present study enabled the assessment of pit and fissure sealing videos not only for educational value but also for source credibility and disclosure-related quality. The positive correlations among the three instruments support the internal consistency of this assessment strategy and suggest that these tools capture overlapping, although not identical, aspects of video quality.

The present findings are consistent with previous studies of health information videos on YouTube, TikTok, and Chinese short-video platforms, where content is often accessible but varies substantially in completeness, reliability, and transparency.^11,39,40^ A recent systematic review of orthodontic content on social media reached a similar conclusion, reporting that informational quality is often low or inconsistent across platforms.^16^ In line with earlier work, highly engaging videos in the present study were not necessarily more reliable or useful, reinforcing the view that platform popularity metrics should not be interpreted as proxies for scientific credibility.^29^

At the same time, this study highlights a concern that may be especially relevant to preventive oral health. Compared with many disease-oriented topics, pit and fissure sealing may appear relatively familiar, simple, and strongly benefit-oriented from a lay perspective. This may facilitate dissemination but also increase the risk of oversimplification. In the present sample, the main issue was not widespread overt misinformation. Rather, it was the selective omission of clinically and practically important topics, especially contraindications, maintenance, and service-related information. For preventive interventions, such partial communication may still shape parental expectations, decision-making, and service uptake.

Another noteworthy finding was the lack of a meaningful association between engagement and validated quality scores.^29^ In the context of children’s preventive care, popular content may appear persuasive while remaining insufficiently comprehensive. This distinction matters because parental decisions are often made in low-threshold information environments, where accessible narratives may exert greater influence than detailed clinical explanations. The present findings, therefore, suggest that, in the context of pit and fissure sealing, informational balance may be as important as factual correctness.

### Public Health Implications

These findings have implications for both oral health communication and service delivery. First, short-video platforms appear to be more effective at raising awareness than at supporting informed decision-making. The frequent emphasis on benefits and indications may increase public familiarity with pit and fissure sealing. However, the underrepresentation of contraindications, maintenance, and service access means that users may still receive only a partial understanding of the intervention.

Second, professional participation in digital oral health communication should be strengthened. Paediatric dentists, preventive dentistry specialists, public hospitals, and professional associations are well-positioned to produce evidence-based, practice-oriented videos. However, reliable content will have a limited public health impact if it remains less visible than simplified, more engaging material. Communication training that helps oral health professionals adapt evidence-based messages to short-form video may therefore be just as important as clinical expertise itself.

Third, platforms may benefit from stronger governance mechanisms for health-related content. Verified professional identity, clearer source labelling, and greater visibility of credible health information may help users distinguish between popular and reliable content. Because engagement metrics were not associated with validated quality scores in this study, visible indicators of credibility may be especially important for users who lack the time or expertise to critically evaluate medical videos. These recommendations are consistent with broader calls to improve the quality of digital health information and reduce exposure to misleading online medical content.^1,8^

Fourth, the findings underscore the importance of digital oral health literacy. Parents and caregivers may understand the general message that pit and fissure sealing is beneficial, yet still lack the information needed to assess suitability, limitations, or follow-up requirements. Public health education should therefore not only promote preventive services, but also improve the public’s ability to identify balanced, source-transparent, and clinically useful information in digital environments.

### Limitations and Future Work

This study has several limitations. First, it was cross-sectional and captured the short-video environment at a single time point; given the dynamic nature of platform content and ranking systems, the observed patterns may not remain stable over time. Second, only one Chinese search term was used to standardise retrieval across platforms. Although this approach improved comparability, it may not have captured the full range of videos that users encounter when searching with alternative professional or colloquial terms. Third, the sample was restricted to Chinese-language videos on TikTok and Bilibili, and the findings may therefore not be generalisable to other platforms, languages, or media settings. Fourth, videos without audio narration were excluded to improve comparability in assessing spoken health communication. However, some short videos rely primarily on captions, on-screen text, or visual demonstrations, and excluding such videos may have introduced selection bias. Fifth, although standardised instruments and a predefined content framework were used, some degree of subjectivity is unavoidable in content coding and quality assessment. Sixth, GQS and mDISCERN are widely used in consumer health information studies, but they were not originally developed for short-video platforms. The brevity, multimodal presentation, and algorithm-mediated visibility of short-video content may affect both how information is communicated and how quality is perceived. Accordingly, results derived from these instruments should be interpreted with caution in this specific media context. Finally, this study focused on video characteristics and informational quality. It did not examine how exposure to such content influences parental knowledge, attitudes, decision-making, or actual uptake of pit and fissure sealing.

Future studies should examine how the content and quality of pit and fissure sealing videos change over time and compare search-based exposure with recommendation-based exposure. It would also be valuable to assess whether platform-level interventions, such as professional verification, expert review labels, or stronger source disclosure, can improve the visibility of high-quality oral health information. In addition, linking online information exposure with parental knowledge, decision-making, and actual sealant utilisation would help clarify the real-world impact of digital preventive communication.

## CONCLUSION

Chinese-language short videos on pit and fissure sealing posted on TikTok and Bilibili provide moderately useful information, but their content remains incomplete and uneven. Most videos emphasise benefits, indications, and basic concepts, whereas clinically important topics such as contraindications, maintenance, and cost are discussed much less often. Videos uploaded by healthcare professionals, especially SHCP, tend to achieve higher quality, reliability, and transparency scores and are more likely to cover clinically relevant content than videos uploaded by IUs. In contrast, user engagement does not reflect informational quality, indicating that popularity should not be interpreted as a marker of medical credibility. These findings underscore the need to strengthen digital oral health communication, encourage greater participation from healthcare professionals, and improve access to balanced, trustworthy, and practical preventive information on short-video platforms.

### Acknowledgements

#### Funding

The authors received no financial support for the research, authorship, and/or publication of this article.

#### Conflict of interest

The authors declare that they have no conflicts of interest related to this study.

#### AI use statement

Generative artificial intelligence was used only to assist with language editing and manuscript polishing. The authors critically reviewed and revised all AI-assisted text and take full responsibility for the accuracy, integrity, and originality of the final manuscript. No AI tool was used to generate, analyse, or interpret the study data. The tool used was ChatGPT.

## Appendix

**Table A1:** Global Quality Score criteria

Score	Description
1	Poor quality; poor flow of the videos; most information missing; not at all useful for patients
2	Generally poor quality; some information listed, but many important topics missing; of very limited use to patients
3	Moderate quality; suboptimal flow; some important information adequately discussed, but other information poorly discussed; somewhat useful for patients
4	Good quality and generally good flow; most of the relevant information listed, but some topics not covered; useful for patients
5	Excellent quality and flow; very useful for patients
Scores range from 1 to 5, with higher scores indicating better overall quality and greater usefulness for patients. GQS, Global Quality Score.	

**Table A2:** mDISCERN criteria

Item	Criterion
1	Is the video clear, concise, and understandable?
2	Are valid sources cited?
3	Is the content presented balanced and unbiased?
4	Are additional sources of content listed for patient reference?
5	Are areas of uncertainty mentioned?
Each item was scored 1 if present and 0 if absent, yielding a total score of 0 to 5. Higher scores indicate better reliability and completeness of health information. mDISCERN, modified DISCERN.	

**Table A3:** JAMA benchmark criteria

Criterion	Description	Score
Authorship	Author and contributor credentials and their affiliations should be provided.	1
Attribution	Clearly lists all copyright information and states references and sources for content.	1
Currency	Initial date of posted content and subsequent updates to content should be provided.	1
Disclosure	Conflicts of interest, funding, sponsorship, advertising, support, and video ownership should be fully disclosed.	1
Each criterion was scored as 1 if present and 0 if absent, yielding a total score ranging from 0 to 4. Higher scores indicate greater adherence to the JAMA benchmark criteria. JAMA, *Journal of the American Medical Association*.		
